# Knowledge and Attitude of Medical Students Towards Helicobacter pylori Infection and Its Prevention and Management: A Study From Riyadh, Saudi Arabia

**DOI:** 10.7759/cureus.51174

**Published:** 2023-12-27

**Authors:** Shahad M Alajmi, Taeef M Alsulami, Munirah A Ben Mudayhish, Maylan A Alhawas, Mona S Alangari, Ali Alfarhan, Aamir Omair

**Affiliations:** 1 College of Medicine, King Saud bin Abdulaziz University for Health Sciences, Riyadh, SAU; 2 Family Medicine/Primary Health Care, Ministry of National Guard - Health Affairs, Riyadh, SAU; 3 Medical Education, College of Medicine, King Saud bin Abdulaziz University for Health Sciences, Riyadh, SAU

**Keywords:** healthcare practitioners’ attitude, helicobacter, infectious diseases, knowledge assessment, medical school students

## Abstract

Background

*Helicobacter pylori (H. pylori*) is a virulent pathogen causing gastritis and ulcers followed by serious complications. Despite being a heavy burden to eradicate, there are not many studies that assess the comprehension of future physicians regarding this bacterium. The objective of this study was to assess medical students’ knowledge and attitude toward *H. pylori *while evaluating the variations based on their socio-demographic factors at King Saud bin Abdulaziz University for Health Sciences (KSAU-HS) in Riyadh, Saudi Arabia.

Methods

A cross-sectional study was conducted among students in all four years of medical college. The data was collected by distributing an online questionnaire which included three following sections: demographic data, knowledge regarding *H. pylori* infection, and attitude toward *H. pylori *infection. Total knowledge and attitude levels were grouped into three and two categories, respectively, and compared between the respondents’ socio-demographics.

Results

Out of 330 respondents, the majority were females (n=185, 56%), and the mean age was 22.8±2.1 years. There were 184 students (56%) who had an excellent attitude (>70%) and 140 (44%) students had average knowledge (34-70%). The medical students' knowledge level was significantly (p<0.001) different between the participants according to their year of study and gender, with higher scores reported by male students in their clinical years (sixth and fifth years).

Conclusion

Medical students of KSAU-HS, Riyadh, had an overall average knowledge and excellent attitude towards *H. pylori* infection, and its prevention and management which emphasize the need for more comprehensive education and awareness programs throughout the medical curriculum to ensure future physicians are well-prepared to address the challenges associated with *H. pylori*-related health issues.

## Introduction

*Helicobacter pylori *(*H. pylori*), a highly widespread pathogen, inhabited around 4.4 billion individuals worldwide [1]. *H. pylori *transmission is not thoroughly comprehended; however, it is thought that person-to-person transmission, including gastro-oral, fecal-oral, and oral-oral, specifically occurring within the family, is the method of infection [2]. Furthermore, its pathogenicity is related to many virulence factors and interactions of the host immune system [1]. Globally, *H. pylori* prevalence ranges from 18.9% to 87.7%, but with improved hygiene practices, the incidence of *H. pylori* infection has been declining [3]. In the Middle East and North Africa (MENA) region, *H. pylori* prevalence ranged between 36.8% and 94% in adults [4]. The incidence of *H. pylori *infection was 34.7% in a study conducted in 2020, in Riyadh, Saudi Arabia [5]. Moreover, for diagnosing *H. pylori* infection, the methods can be categorized into invasive and non-invasive methods [6]. To illustrate, an invasive method includes endoscopy such as white light imaging (WLI), and non-invasive methods such as rapid urease test (RUT) and urea breath test (UBT) [7,8]. Quadruple or triple therapies are known regimens for *H. pylori *that consider factors such as resistance to effectively eradicate this bacterium [9].

*H. pylori* infection is a significant etiological agent for various gastric complications, such as peptic or duodenal ulcers and gastritis [10,11]. *H. pylori* is highly associated with gastric and duodenal ulcers because of its ability to resist the gastric acid that is normally protective against a wide range of bacteria products [12]. *H. pylori* infection is also associated with a wide range of non-gastrointestinal diseases. *H. pylori* has a special molecular mimicry with the host nervous tissue; therefore, it could lead to diverse neurodegenerative diseases [13]. In individuals with post-hepatic encephalopathy, it can exacerbate their symptoms causing cognitive impairment which is related to falls and fractures in patients with cirrhosis [13]. *H. pylori* can also contribute to the pathophysiology of iron deficiency anemia, and it is related to different metabolic syndromes including diabetes, dyslipidemia, hypertension, cardiovascular disease, and non-alcoholic fatty liver disease [13]. It is well established that alcohol consumption, poor diet, and poor knowledge regarding *H. pylori* infection transmission are preventable risk factors for *H. pylori* infection dissemination [14].

Despite the high prevalence of *H. pylori* infection, there are not enough studies that assess the knowledge and attitude of students or general practitioners towards this bacterium. Jukic et al. showed that even those with higher medical comprehension and clinical experience can still lack the knowledge that is related to treatment and antibiotic resistance [15]. Locally, in Saudi Arabia, a study was conducted at King Saud University, Riyadh, to compare the *H. pylori* knowledge between health science and non-health science college students [16]. In both the two undergraduate groups, less than 10% had good knowledge [16].

Considering that health professionals play a central role in medical science and preventive measures against the risk caused by a wide variety of bacteria, evaluating the medical students' understanding can contribute to enhancing healthcare. The objective of this study was to measure the knowledge and attitude of medical students at King Saud bin Abdulaziz University for Health Sciences (KSAU-HS) in Riyadh regarding *H. pylori* infection, prevention, and management while evaluating the differences depending on their socio-demographics.

## Materials and methods

Study design, setting, and participants

In this cross-sectional study, a questionnaire-based survey was conducted between September 2021 and March 2022. The study was performed in KSAU-HS, College of Medicine, Riyadh, Saudi Arabia. The participants involved in this study were all enrolled medical students in KSAU-HS in Riyadh. The research included female and male medical students from both academic stream I (who graduated from high school) and academic stream II (who already obtained their bachelor's degree in scientific fields and are studying medicine as a second major). Furthermore, batches 15, 16, 17, and 18 represent sixth-year, fifth-year, fourth-year, and third-year medical students, respectively, were included. Sixth-year and fifth-year students are in their clinical years, while fourth-year and third-year students represent pre-clinical students. However, batches 19 and 20 were excluded since they were pre-med students. Students were recruited using a non-probability convenience sample technique as the study included all accessible students who were willing to participate. The sample size was 294 medical students which was calculated out of 1240 medical students with a confidence level of 95% and a 5% margin of error using the Raosoft sample size calculator (Seattle, WA: Raosoft, Inc.) [17].

Data collection tool and process

Data was collected by distributing a self-administered, online survey in the English language using Google Forms. Some of the relevant questions and statements were obtained from four different studies, with some adjustments to the format to meet the research objectives, while the others were developed by the authors [18-21]. Two experts checked the questionnaire's validity and slight adjustments were made according to their recommendations. In the survey, a question regarding the agreement to participate in the study was attached and considered as informed consent along with completing the whole questionnaire. Moreover, students were voluntary and free to withdraw at any time.

The three sections of the questionnaire included demographic data, knowledge regarding *H. pylori* infection, and finally attitude toward *H. pylori* infection (tables in appendices). The first section had five questions regarding the demographic data of the participants. The second section had 12 questions focusing on the knowledge of *H. pylori* infection, prevention, and management. Participants had to read each question and respond to it using a three-point scale (yes/no/do not know). The attitude section included 10 statements regarding the infection, prevention, and management of *H. pylori*, in a five-point Likert Scale format (strongly agree, agree, neutral, disagree, and strongly disagree).

Statistical analysis

All data were entered after coding them in Microsoft Excel and then were cleaned and transferred to SPSS version 20 statistical software (Armonk, NY: IBM Corp.) to analyze and implement the descriptive statistics. The Cronbach’s alpha for the attitude section was 0.68, and it was 0.83 for the knowledge section. Students’ knowledge and attitude were considered as the outcome (dependent) variables, whereas grouping variables (independent) involved the characteristics of the participants which are age, gender, batch (year of study), academic stream, and income. Numerical data which are age, total knowledge, and attitude scores were presented as mean and standard deviation, and skewed data were presented as a median and interquartile range (IQR). In addition, categorical data which are students’ gender, academic stream, batch, income level, knowledge level, and attitude level were presented as frequencies and percentages. 

The correct answer was awarded one point, and the incorrect answer was scored as zero; therefore, the maximum score was 12 and the minimum score was 0 in the second part of the questionnaire regarding knowledge about *H. pylori* infection. In the third section of the questionnaire regarding attitude toward *H. pylori* infection, the maximum score was 50 and the minimum score was 10. The knowledge and attitude were categorized into poor, average, and excellent. The cut-off scores were poor (0-33%), average (34-70%), and excellent (>70%) for both knowledge and attitude. The latter was recategorized into two groups, average (up to 70%) and excellent (>70%) since very few respondents had poor attitudes which could negatively affect the data.

Chi-square test of association was employed to evaluate variations in knowledge and attitude among distinct socio-demographic groups. Other tests such as two independent sample t-test and one-way ANOVA were applied as applicable to further assess the numerical differences in the knowledge section. The statistical test was considered significant if the p-value was less than 0.05 for all of the tests applied.

Ethical consideration

Ethical approval was obtained by the Institutional Review Board (IRB) of King Abdullah International Medical Research Center (KAIMRC) with ref no. IRBC/0968/21. No personal identification was taken from the participants, and data was secured and only accessible to the research team. Anonymity and confidentiality were protected during the data collection until the end of the study.

## Results

Socio-demographic characteristics

There was a total of 330 students who responded to the survey; most of the respondents were females (n=185, 56%), with a mean age of 22.8±2.1 years, and students of academic stream I (n=291, 88%), with the highest proportion are in their third year (n=119, 36%). There were 201 (61%) students with a family income of more than 20,000 Saudi Riyals per month (Table 1).

**Table 1:** Socio-demographic characteristics of the respondents (n=330). *Stream I are persons who studied medicine after they graduated from high school and stream II are persons who already obtained their bachelor’s degree in scientific fields and are studying medicine as a second major. SAR: Saudi Riyal

Items		n (%)
Gender	Male	145 (44%)
	Female	185 (56%)
Age	Mean±SD	22.8±2.1
Year of study	6th year	49 (15%)
	5th year	52 (16%)
	4th year	110 (33%)
	3rd year	119 (36%)
Streams*	Stream I	291 (88%)
	Stream II	39 (12%)
Income (SAR)	<5,000	11 (3%)
	5,000-9,999	19 (6%)
	10,000-14,999	51 (15%)
	15,000-19,999	48 (15%)
	20,000+	201 (61%)

The total possible score of the knowledge section was 12, and medical students had a mean score of 6.8±3.4. Students were rewarded 1 score for correct answers and 0 for incorrect ones, giving a maximum score of 1 for each question in the knowledge section which had 12 questions. The knowledge of medical students that “Do duodenal or gastric ulcers result from *H. pylori *infection?” had the highest mean (0.7±0.4) as well as “Do *H. pylori* cause burring ache and indigestion?” with a mean of (0.7±0.4). However, the lowest mean score (0.3±0.4) was recorded regarding the question “Can an infected person with *H. pylori* transmit the infection through body fluids?” with 108 (33%) of medical students answering it correctly (Table 2).

**Table 2:** Number of correct answers for individual questions of the knowledge section (n=330)

Questions/items	Correct answers n (%)
Can consumption of contaminated food transmit *H. pylori* bacteria?	227 (69%)
Can an infected person with *H. pylori* transmit the infection through body fluids?	108 (33%)
Does *H. pylori* infection resolve spontaneously?	166 (50%)
Does gastric cancer result from *H. pylori* infection?	185 (56%)
Do duodenal or gastric ulcers result from *H. pylori* infection?	241 (73%)
Does *H. pylori* infection cause burning aches and indigestion?	237 (72%)
Does obtaining a small tissue sample from the stomach using endoscopy detect *H. pylori* bacteria?	208 (63%)
Can *H. pylori* infection be diagnosed by urea breath test (UBT)?	191 (58%)
Can *H. pylori* infection be diagnosed by serology tests?	136 (41%)
Can drinking yogurt treat *H. pylori* infection?	142 (43%)
Is there any effective treatment for *H. pylori* infection?	230 (70%)
As a first-line therapy, triple therapy is used to eradicate* H. pylori* infection?	177 (54%)

Medical students reported a mean of 38.9±5.4 in the attitude section which was formatted on a five-point Likert scale with a maximum total score of 50. Answering with strongly agree was rewarded a maximum score of 5 for individual statements. The attitude statement with the highest mean score (4.4±1) was “Hand sanitation is important before eating to prevent *H. pylori *infection." While the lowest mean (2.8±1.25) was attributed to the statement “I think regular screening for *H. pylori* is useful” with many students, 110 (33%), choosing neutral as an answer for this statement (Table 3).

**Table 3:** Mean score* for individual statements of the attitude section (n=330). *Answering with strongly agree was rewarded a maximum score of 5 for individual statements.

Statement	Mean score
Hand sanitation is important before eating to prevent *H. pylori* infection.	4.4±1
Hand sanitation is important after toileting to prevent *H. pylori* infection.	4.3±1
I think that if a person’s dietetic hygiene is poor, he/she may get affected by *H. pylori* bacteria.	4.1±1
I think regular screening for *H. pylori* is useful.	2.8±1.25
I think that combining non-invasive tests, such as urea breath test, and invasive tests, such as rapid urease test, are more efficient to establish the diagnosis of *H. pylori* bacteria.	3.7±1
I believe that eliminating *H. pylori* bacteria reduces the risk of developing gastric cancer.	4.1±0.9
As a future physician, I am worried about *H. pylori* bacteria resistance.	4±1
I think there is no effective treatment for *H. pylori* infection.	3.9±1
I expect that triple therapy for treating *H. pylori* infection as a first-line treatment is better than quadruple therapy in terms of effectiveness, compliance, and side effects.	3.6±0.9
I believe that patient adherence to the multidrug regimen is the main determinant of successful treatment.	4.3±0.86

**Figure 1:**
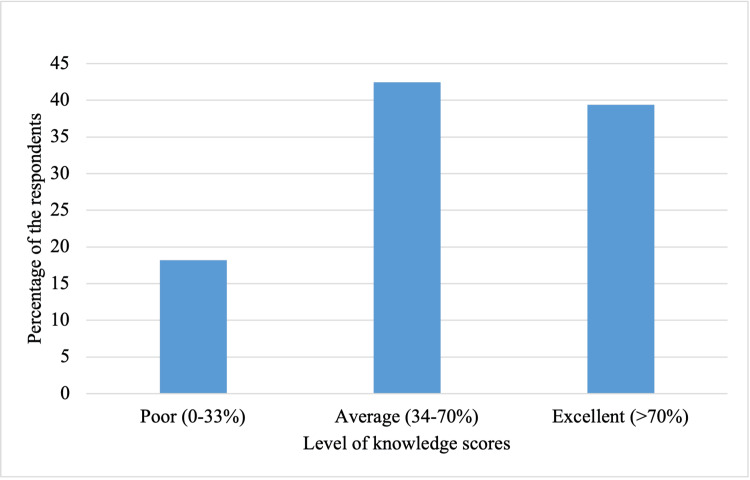
Medical students' knowledge level regarding H. pylori.

Out of 330 respondents, 140 students (42%) had an average knowledge level scoring between 34% and 70%. Students who achieved excellent scores (>70%) were 130 (41%), and 60 (17%) students had poor (0-33%) knowledge (Figure 1).

**Figure 2:**
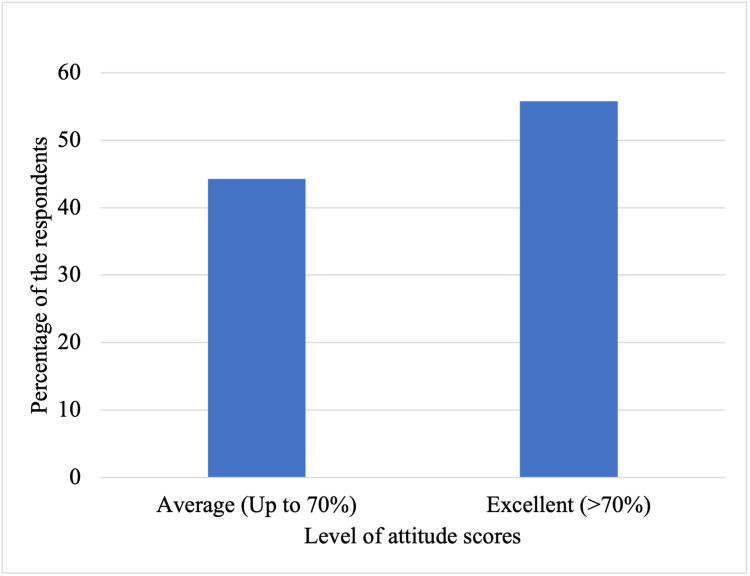
Medical students' attitude level regarding H. pylori.

Regarding the attitude section, there were 184 (56%) medical students who had an excellent attitude (>70%) regarding *H. pylori* infection, prevention, and management. On the other hand, 146 (44%) had an average (up to 70%) attitude (Figure 2).

Differences in the knowledge level of the participants

Chi-square test of association demonstrated that the level of students’ knowledge regarding *H. pylori *was significantly different between the participants depending on their academic year and gender (p<0.001). However, there was no significant difference between the knowledge level between streams I and II (p=0.06) (Table 4).

**Table 4:** Differences in the knowledge level of the participants according to their socio-demographics (n=330). Chi-square test of association was performed. *A p-value below 0.05 indicates statistically meaningful disparities in knowledge levels. SAR: Saudi Riyal

Socio-demographic data		Level of knowledge regarding *H. pylori* infection			
		Poor (0-33%), n (%)	Average (34-70%), n (%)	Excellent (>70%), n (%)	p-Value
Gender	Male	12 (8%)	58 (40%)	75 (52%)	<0.001^*^
	Female	48 (26%)	82 (44%)	55 (30%)	
Year of study	6th year	0	16 (33%)	33 (67%)	<0.001^*^
	5th year	0	22 (42%)	30 (58%)	
	4th year	30 (27%)	41 (37%)	39 (36%)	
	3rd year	30 (25%)	61 (51%)	28 (24%)	
Stream	Stream 1	54 (19%)	129 (44%)	108 (37%)	0.06
	Stream 2	6 (15%)	11 (28%)	22 (56%)	
Income (SAR)	<5,000	2 (18%)	6 (55%)	3 (27%)	0.90
	5,000-9,999	3 (16%)	11 (58%)	5 (26%)	
	10,000-14,999	9 (18%)	21 (41%)	21 (41%)	
	15,000-19,999	10 (21%)	18 (38%)	20 (42%)	
	20,000+	36 (18%)	84 (42%)	81 (40%)	

When two independent sample t-tests were applied, there was a statistically significant gender difference in the knowledge level (p<0.01) with male students having a higher (7.8±2.9) mean than females (6.04±3.6) (Table 5).

**Table 5:** Difference of total knowledge scores according to the participants’ gender and stream (n=330). Two independent sample t-tests were applied. *A p-value below 0.05 indicates statistically meaningful disparities in knowledge levels.

Socio-demographic		n	Mean±SD	p-Value
Gender	Male	145	7.8±2.9	<0.01*
	Female	185	6.0±3.6	
Stream	Stream 1	291	6.7±3.4	0.70
	Stream 2	39	7.8±3.4	

In addition, one-way ANOVA was applied to the year of study and income. Knowledge level was significantly different between the students according to their year of study (p<0.01). Sixth-year and fifth-year students had means of 9.2±1.5 and 8.9±1.7, respectively. Also, students in their fourth year scored a mean of 6.0±3.8, whereas a slightly lower mean of 5.6±3.2 was scored by third-year students (Table 6).

**Table 6:** Difference of total knowledge scores according to the participants’ year of study and income (n=330). One-way ANOVA was applied. *A p-value below 0.05 indicates statistically meaningful disparities in knowledge levels. SAR: Saudi Riyal

Socio-demographic		n	Mean±SD	p-Value
Year of study	6th year	49	9.2±1.5	<0.01*
	5th year	52	8.9±1.7	
	4th year	110	6.0±3.8	
	3rdyear	119	5.6±3.2	
Income (SAR)	<5,000	11	6.2±3.3	0.83
	5,000-9,999	19	6.1±3.4	
	10,000-14,999	51	6.9±3.6	
	15,000-19,999	48	6.7±3.4	
	20,000+	201	6.9±3.4	

Differences in the attitude level of the participants

Chi-square test of association application on the medical student’s socio-demographics showed no significant difference between the participants regarding their socio-demographics (Table 7).

**Table 7:** Differences in the attitude level of the participants according to their socio-demographics (n=330). Chi-square test of association was performed. A statistically significant p-value was defined as below 0.05. SAR: Saudi Riyal

Socio-demographic		Level of attitude regarding *H. pylori* infection		
		Average (up to 70%) n (%)	Excellent (>70%) n (%)	p-Value
Gender	Male	61 (42%)	84 (58%)	0.48
	Female	85 (46%)	100 (54%)	
Year of study	6th year	20 (41%)	29 (59%)	0.32
	5th year	28 (54%)	24 (46%)	
	4th year	51 (46%)	59 (54%)	
	3rd year	47 (39%)	72 (61%)	
Stream	Stream 1	130 (45%)	161 (55%)	0.66
	Stream 2	16 (41%)	23 (59%)	
Income (SAR)	<5,000	8 (73%)	3 (27%)	0.21
	5,000-9,999	9 (47%)	10 (53%)	
	10,000-14,999	21 (41%)	30 (59%)	
	15,000-19,999	25 (52%)	23 (48%)	
	20,000+	83 (41%)	118 (59%)	

## Discussion

This cross-sectional study was carried out to determine medical students' level of knowledge and attitude toward *H. pylori* infection, prevention, and management while assessing the differences according to their socio-demographics. The medical students’ overall knowledge level was average, whereas their attitude level was excellent, and male students in their clinical years fifth and sixth demonstrated higher knowledge levels (p<0.001) compared to other participants.

In this study, the knowledge and attitude levels of students cannot be solely attributed to their year of study. In general, students achieved higher mean scores on the questions “Does gastric cancer result from *H. pylori* infection?” and “Does *H. pylori *infection cause burning ache and indigestion?”. These questions were correctly answered by the general population in a study conducted in Saudi Arabia [22]. This could be attributed to the fact that *H. pylori* is widely recognized for causing gastrointestinal symptoms and manifestations. Nevertheless, one study conducted on the Chinese public and healthcare physicians revealed a substantial disparity in knowledge regarding *H. pylori* symptoms between the general public and healthcare practitioners [23]. Given the prior findings regarding *H. pylori* knowledge discrepancies between the general population and health physicians, it can be assumed that a disparity may exist between the general population and medical students. The question that recorded the lowest mean score among students in different batches (third year to sixth year) was “Can an infected person with *H. pylori* transmit the infection through body fluids?”. This can be explained by the fact that medical students generally lack clinical experience at this stage, which may lead to uncertainty regarding specific medical information. The two studies showed that people generally tend to have limited awareness regarding *H. pylori* route of transmission, whereas physicians, including non-gastroenterologists, have decent knowledge about this topic [22,23]. The attitude statements that had higher mean scores were primarily associated with individuals’ perspectives toward infectious diseases, such as the statement “Hand sanitation is important before eating to prevent *H. pylori* infection.” In another recent Chinese study that targeted the people's awareness, the previous statement was properly answered by the general public [24]. On the other hand, attitude statements with diagnostic and treatment focus, such as “I think regular screening for *H. pylori* is useful,” were the ones that challenged the students in this study. However, some general practitioners who were actively engaged in clinical settings may have insufficient knowledge of *H. pylori *diagnosis and treatment recommendations based on a study done by Cano-Contreras et al. [25]. Therefore, considering what was mentioned previously, educational measurements should be taken to not only improve the students’ knowledge but also to improve the healthcare provided by future physicians.

When considering different batches’ knowledge levels, batch 15 (sixth-year medical students) had the highest level of knowledge. This could be attributed to the fact that they completed an entire course about gastrointestinal infections, engaged in clinical rotations, and had an overall more extensive experience in the medical field compared to the other batches. Additionally, batch 18 (third-year medical students) had the lowest level of knowledge. This could be explained by the fact that they had recently started studying in-depth basic medical knowledge and had not yet undergone a course that involved *H. pylori* infection. Likewise, a study was done at King Saud University among both health science and non-health science students in Riyadh, Saudi Arabia, and it showed that the respondents’ knowledge level was significantly associated with their university level [16]. In regards to gender, male students displayed higher knowledge levels by correctly answering more questions compared to female students. The increased knowledge level among male students could be due to the fact that most respondents were male students from batch 15 (sixth-year medical students) in comparison with female students from other batches. However, a recent study focused on people’s knowledge about *H. pylori* found that the awareness level was significantly higher in females who constituted the majority of respondents [26]. Thus, the knowledge level difference between the two genders cannot be ensured yet with these disparities in the number of respondents. Moreover, there was a study conducted in Al-Ahsa demonstrated that there was no significant difference between females' and males’ knowledge levels regarding *H. pylori* [27].

Regarding the other demographic characteristics of the participants in this study, there was no significant difference observed in terms of the participants’ academic stream since the curriculum is uniform for both streams and considering the fact that topics such as *H. pylori* infection and management are rarely thoroughly taught in any other specialty. For socioeconomic status, students study for free as KSAU-HS is a governmental university that provides unlimited free access to some educational platforms and libraries. This may explain why income level was insignificantly associated with knowledge level. However, a systematic review demonstrated that higher socioeconomic status is one of the factors that are well-established to protect against *H. pylori* infection [28]. Another study done in China showed that low income is one of the factors that is associated with *H. pylori* infection recurrence [29]. Although the two studies above can show socioeconomic status as a protective or predisposing factor to *H. pylori* infection, it is important to recognize that many factors beyond peoples’ knowledge in poor or wealthy populations can contribute to this association, such as crowded living conditions, inadequate hygiene practices, and untreated water sources.

Limitations

Being one of the few studies comparing knowledge and attitude levels among medical students regarding *H. pylori* infection, management, and prevention in Saudi Arabia is the point of strength of this study. However, since this study was conducted in one university, the generalizability of the results is a limitation. Another limitation of this study is that convenience sampling method was applied; therefore, the recruitment of the respondents was based on the accessibility and availability of the participants which might lead to sampling bias. In addition, the study did not assess the sources from which medical students acquire information, such as textbooks, articles, online resources, and question banks, which can have an impact on their knowledge and attitude levels. Moreover, this study did not inquire about participants’ previous history of having *H. pylori* infection, which could potentially affect the findings as individuals with previous* H. pylori* infection who experienced the symptoms and consulted healthcare professionals could obtain information regarding the infection. Furthermore, previous research found that the students' knowledge level regarding *H. pylori* was significantly associated with earlier infection of this organism [16]. Indeed, further studies should consider exploring these aspects with an appropriate and extensive sample size to provide a more comprehensive understanding of the factors affecting knowledge and attitude levels of *H. pylori* infection.

## Conclusions

This study found that KSAU-HS Riyadh medical students displayed an overall average knowledge level and excellent attitude level toward *H. pylori *infection. Notably, knowledge levels of clinical year students were higher than pre-clinical year students, and gender difference was significant with male students scoring higher in this section. Implementing medical education campaigns on *H. pylori *infection can enhance students’ knowledge and public awareness, supporting early detection and intervention through screening programs. These findings can inform the development of new educational programs to boost awareness and knowledge about *H. pylori *infection. Larger-scale studies should further investigate these aspects for a more comprehensive understanding.

## Appendices

The research's data collection tool, the questionnaire, is presented below. The socio-demographic questions addressed to the respondents are displayed in Table 8. Medical students' knowledge regarding *H. pylori *was collected by asking the following questions in Table 9. The final section of the questionnaire was directed toward the medical students' attitudes about *H. pylori* infection (Table 10).

**Table 8:** The socio-demographic questions addressed to the respondents.

No.	Questions	Response
1	What is your gender?	Male
		Female
2	How old are you? (Open question)	
3	Which batch are you in?	15
		16
		17
		18
4	Which stream are you in?	Stream 1
		Stream 2
5	What is your family income level (SAR)?	<5,000
		5,000-9,999
		10,000-14,999
		15,000-19,999
		>20,000

**Table 9:** Knowledge of H. pylori infection, prevention, and management.

No.	Questions	Response
6	Can consumption of contaminated food transmit *H. pylori* bacteria?	Yes
		No
		Do not know
7	Can an infected person with *H. pylori* transmit the infection through body fluids?	Yes
		No
		Do not know
8	Does *H. pylori *infection resolve spontaneously?	Yes
		No
		Do not know
9	Does gastric cancer result from *H. pylori* infection?	Yes
		No
		Do not know
10	Do duodenal or gastric ulcers result from *H. pylori* infection?	Yes
		No
		Do not know
11	Does *H. pylori* infection cause burning ache and indigestion?	Yes
		No
		Do not know
12	Does obtaining a small tissue sample from the stomach using endoscopy detect *H. pylori* bacteria?	Yes
		No
		Do not know
13	Can *H. pylori* infection be diagnosed by urea breath test (UBT)?	Yes
		No
		Do not know
14	Can *H. pylori *infection be diagnosed by serology tests?	Yes
		No
		Do not know
15	Can drinking yogurt treat *H. pylori* infection?	Yes
		No
		Do not know
16	Is there any effective treatment for *H. pylori* infection?	Yes
		No
		Do not know
17	As a first-line therapy, triple therapy is used to eradicate *H. pylori* infection?	Yes
		No
		Do not know

**Table 10:** Attitudes towards H. pylori infection, prevention, and management.

No.	Statements	Response
18	Hand sanitation is important before eating to prevent *H. pylori *infection.	Strongly agree
		Agree
		Neutral
		Disagree
		Strongly disagree
19	Hand sanitation is important after toileting to prevent *H. pylori* infection.	Strongly agree
		Agree
		Neutral
		Disagree
		Strongly disagree
20	I think that if a person’s dietetic hygiene is poor, he/she may get affected by *H. pylori* bacteria.	Strongly agree
		Agree
		Neutral
		Disagree
		Strongly disagree
21	I think regular screening for *H. pylori* is useful.	Strongly agree
		Agree
		Neutral
		Disagree
		Strongly disagree
22	I think that combining non-invasive tests, such as urea breath test, and invasive tests, such as rapid urease test, are more efficient to establish the diagnosis of *H. pylori* bacteria.	Strongly agree
		Agree
		Neutral
		Disagree
		Strongly disagree
23	I believe that eliminating *H. pylori* bacteria reduces the risk of developing gastric cancer.	Strongly agree
		Agree
		Neutral
		Disagree
		Strongly disagree
24	As a future physician, I am worried about *H. pylori* bacteria resistance.	Strongly agree
		Agree
		Neutral
		Disagree
		Strongly disagree
25	I think there is no effective treatment for *H. pylori* infection.	Strongly agree
		Agree
		Neutral
		Disagree
		Strongly disagree
26	I expect that triple therapy for treating *H. pylori* infection as a first-line treatment is better than quadruple therapy in terms of effectiveness, compliance, and side effects.	Strongly agree
		Agree
		Neutral
		Disagree
		Strongly disagree
27	I believe that patient adherence to the multidrug regimen is the main determinant of successful treatment.	Strongly agree
		Agree
		Neutral
		Disagree
		Strongly disagree
